# Anovaginal distance and obstetric anal sphincter injury: a prospective observational study

**DOI:** 10.1007/s00192-018-3838-5

**Published:** 2018-12-10

**Authors:** Sofia Pihl, Eva Uustal, Marie Blomberg

**Affiliations:** Department of Obstetrics and Gynecology, and Department of Clinical and Experimental Medicine, Linköping University, SE-581 85 Linköping, Sweden

**Keywords:** Anovaginal distance, Obstetrical anal sphincter injury, Perineal laceration, Transperineal ultrasound

## Abstract

**Introduction and Hypothesis:**

No measurements are available for diagnosing the extent of obstetric lacerations. The primary aim of this study was to evaluate the relation between the anovaginal distance (AVD) measured with transperineal ultrasound immediately after delivery and external anal sphincter injury. A secondary aim was to assess whether the palpated perineal thickness was associated with the AVD.

**Methods:**

A prospective observational study of 150 primiparous women at the University Hospital, Linköping, Sweden. After vaginal delivery, initial inspection and palpation of the perineal thickness were performed by the midwife. The women were then divided into subgroups depending on the degree of the suspected perineal laceration. Transperineal ultrasound of the AVD was performed by a physician. Diagnostics of the perineal laceration were done according to standard care.

**Results:**

Women with an external sphincter injury had a shorter AVD and shorter palpatory perineal thickness compared with women without anal sphincter injury. No external sphincter injuries were diagnosed when the AVD and/or palpation height was &gt; 20 mm. The mean AVD in the group with probable second-degree laceration (*n* = 85) was 18.8 mm (95% CI 17.8–19.8), in suspected third-degree laceration (*n* = 33) 15.7 mm (95% CI 13.7–17.7) and in probable third-degree laceration (*n* = 32) 11.8 mm (95% CI 9.7–13.9) (*p* &lt; 0.001).

**Conclusions:**

A short AVD could be a warning sign postpartum and should increase the awareness of possible external sphincter injury before suturing. An AVD of 20 mm seems to indicate a cutoff level of the occurrence of external sphincter injury, but this needs further evaluation.

**Electronic supplementary material:**

The online version of this article (10.1007/s00192-018-3838-5) contains supplementary material, which is available to authorized users.

## Introduction

Lacerations of the vagina and perineum after a vaginal delivery are common [2]. Distinguishing an isolated perineal laceration from an anal sphincter injury in the acute phase has to be performed carefully and with high diagnostic consistency at all times to prevent long-term complications of an undiscovered sphincter injury [1]. The risk factors for anal sphincter injury include maternal, delivery and infant characteristics [5]. Short perineal body length is a known maternal risk factor for sphincter injury [4].

Studies have shown that occult sphincter injuries exist as an unidentified anal sphincter tear, insufficient repair of a correctly diagnosed injury or tear of the sutures after repair of the injury [1, 11].Standard care of obstetrical lacerations in most delivery units in Sweden involves an initial inspection and palpation of the vagina, perineum and anal sphincter by the midwife responsible for the delivery. In daily practice, there are no objective criteria for when the obstetrician should be consulted and no other evidence-based diagnostic tools or methods for diagnosing the extent of the obstetric laceration than inspection and palpation. This allows for great variation in practice.

Ultrasound in different forms has emerged as a method of investigating the pelvic floor [12]. Vaginal ultrasound is used by gynecologists and obstetricians on a daily basis during gynecological examinations, but initial examination of obstetrical lacerations with ultrasound is not standard procedure in Sweden, although ultrasound machines are widely available in delivery wards.

Measuring the anovaginal distance (AVD) with transperineal ultrasound using a vaginal probe has shown high interobserver agreement and a short learning curve [9]. The significance of AVD has not yet been evaluated for obstetrical lacerations.

Studies have shown a good correlation between digital perineal palpation and transperineal ultrasound after laceration repair to assess the surgical result [13] and to evaluate the anal sphincter complex after surgery [8, 15]. Whether there is a relation between the extent of the perineal laceration and the perineal thickness measured by palpation directly postpartum and confirmed by transperineal ultrasound of the AVD has not been studied before.

The primary aim of the present study is to evaluate whether there is a relation between the AVD measured with transperineal ultrasound after delivery and diagnosed external anal sphincter injury. A secondary aim was to assess whether the midwives’ initial bidigital examination of the perineum was correlated with the AVD measured with transperineal ultrasound.

## Materials and methods

### Design

This was a prospective observational study of primiparous women.

### Population

The target population was women who had their firstborn child through vaginal delivery at the delivery unit at the University Hospital in Linköping, Sweden, from October 2014 to January 2016. The delivery ward is a tertiary referral center with 3000 deliveries per year. To meet the inclusion criteria of the study, the midwife had to suspect that the woman had a second- or third-degree perineal laceration according to the International Classification of Disease (ICD)-10. First- and fourth-degree perineal lacerations were not included since we assumed that anal sphincter injuries are rare (first degree) or easily detected (fourth degree). Women who met the inclusion criteria could be invited to participate in the study.

The exclusion criteria were inability to understand Swedish or earlier perineal surgery or trauma. In total, 1399 women met the inclusion criteria; of these, 292 women were asked about inclusion and 150 were included in the study after verbal informed consent had been received. A flow chart of the study procedure is presented in Fig. S1. The study population’s age, BMI, smoking habits and proportion living with a partner did not differ from the women who met the inclusion criteria (target population) but were not included (Table 1). The gestational week at delivery was slightly but statistically significantly higher in the study group compared with the target population, although the difference was not clinically relevant.

**Table 1:** Maternal and obstetrical characteristics of primiparous women with suspected grade 2–3 obstetric lacerations

	Study population (*n* = 150)	Target population (*n* = 1399)	*p* value
Age, mean (SD)	29.79(4.22)	29.41 (4.68)	0.19
Body mass index, mean (SD)	24.16 (4.24)	24.48 (4.48)	0.43
Gestational week, mean (SD)	39.96 (1.41)	39.50 (1.87)	0.04
Smoking in early pregnancy	2	55	na
Living with a partner	140	1161	0.17*

*Pearson chi-square test = 2, 1, df 1

## Methods

This study was carried out within clinical practice around the clock. Standard immediate postpartum care in the delivery suite at the clinic is that the woman undergoes an initial inspection and palpation of the obstetric perineal laceration by the midwife responsible for the delivery. The examination consists of a bidigital palpation with one finger at the bottom of the perineal laceration and one finger in the anal canal. Only the distal phalanx of one index finger is inserted in the anal canal, and the other index finger’s distal phalanx is put on the distal posterior vaginal wall. The thickness between the fingertip pads is then assessed. The palpatory thickness of the perineal tissue and observed grade of integrity of the external and internal anal sphincter are noted, and the obstetrician is called if an anal sphincter injury is suspected or if the extent of the perineal laceration is uncertain.

Women thus found to have a suspected second- or third-degree perineal laceration were eligible for the study.

In women who agreed to participate in the study, the midwives were instructed to decide on an initial diagnosis of the extent of the obstetrical laceration after the examination. Alternatives were “probable degree 2,” “suspected degree 3″ or “probable degree 3.” The midwive’s decision was documented in the study protocol. The initial diagnosis of the lacerations according to the responsible midwife was probable degree 2 (*n* = 85), suspected degree 3 (*n* = 33) and probable degree 3 (*n* = 32).

The assessment of the bidigital palpation of the perineal thickness in millimeters was also documented in the study protocol. The midwives who first examined the perineal laceration made an estimation of the palpated perineal thickness and were asked to indicate their assessments in the closest 5-mm intervals.

The women were then examined by a specially educated obstetrician, who was blinded to the midwife’s initial diagnosis and palpated perineal thickness.

The obstetrician performed a standardized examination with transperineal ultrasound, measuring the AVD, and documented the result in the medical record.

The final diagnosis of the extent of perineal laceration was established by the obstetrician according to standard procedure, inspection and palpation, but with the results of the ultrasound examinations available. The laceration was repaired according to the clinical guidelines.

In preparation for the study, all obstetricians (*n* = 23) at the delivery ward were taught how to carry out transperineal ultrasound of obstetrical lacerations by two experienced examiners. The educations entailed co-measurement of the AVD in recruited voluntary women who were newly delivered until the measurements found by the expert and the examiner (± 5 mm) were the same (mean four examinations, range 3–5). This education program is described in detail in a previous article establishing the interobserver agreement of the AVD [9].

**Fig. 1:**
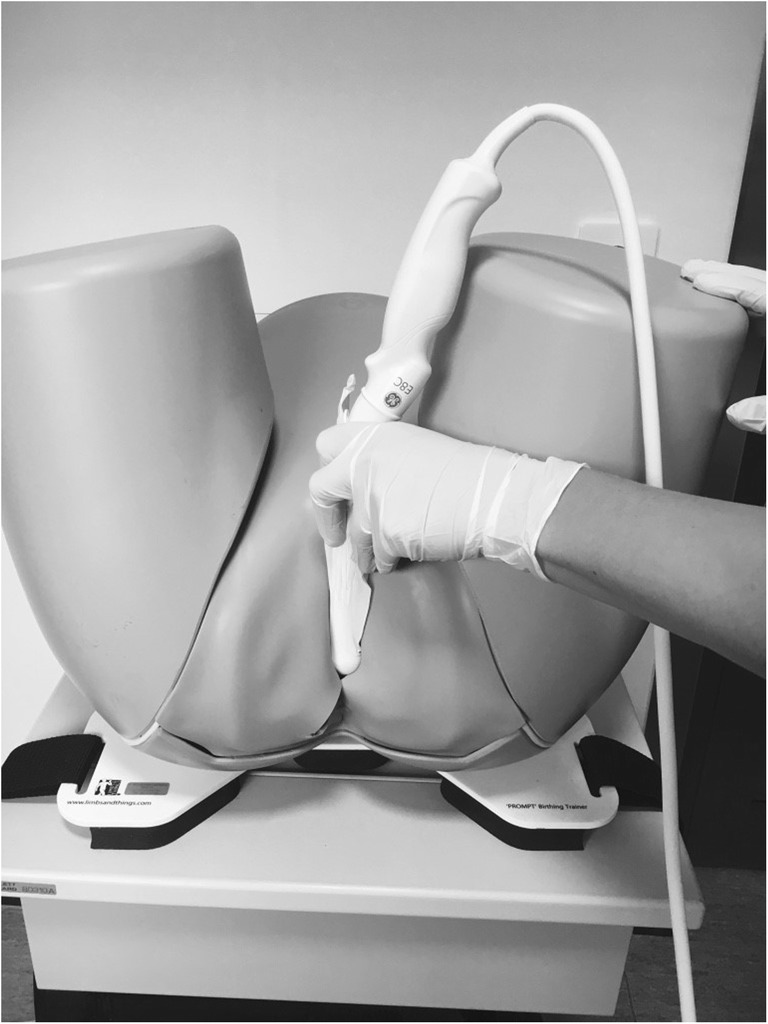
Measuring the anovaginal distance with a vaginal probe

**Fig. 2:**
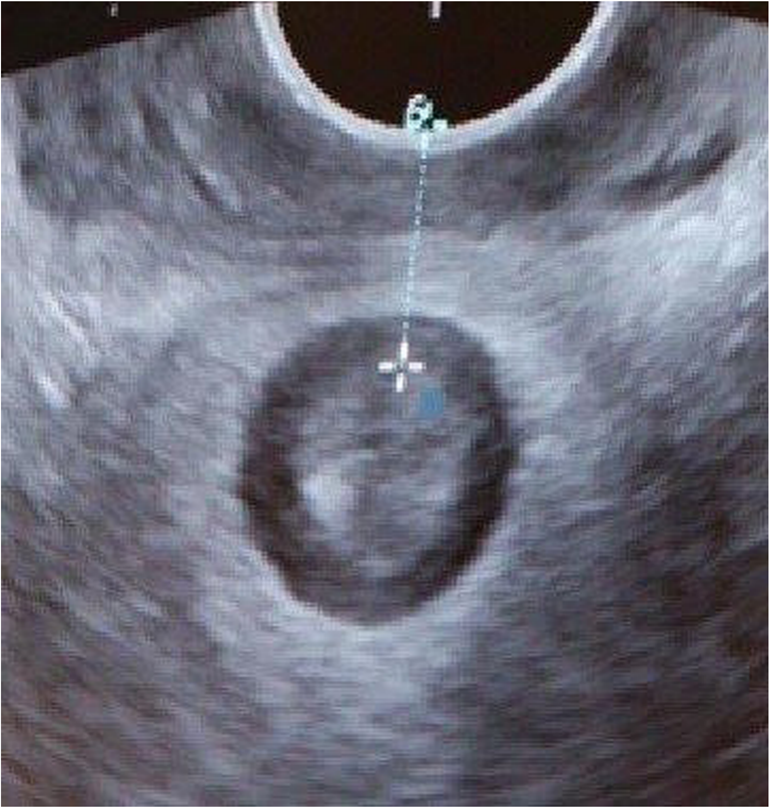
Perineal ultrasound image of the anovaginal distance measured from the anal mucosa to the posterior vaginal wall

AVD measurement by transperineal ultrasound was performed in a standardized procedure. The examination was done with the woman in the lithotomy position. The ultrasound machine used was a Voluson Station with a 9–5 MHz vaginal scanner, type E8C. The probe was placed at a right angle to the posterior vaginal distal wall and in a transverse scanning plane, as seen in Fig. 1. The probe was moved cranially in the vagina from the distal to middle level anal canal until the internal anal sphincter could be seen as a low-echogenic ring. The distance between the anal edge of the internal sphincter and the probe was measured (Fig. 2). In cases with internal sphincter injury, the distance between the inner edge of the anal mucosa and vaginal probe was measured. This distance was defined as the AVD and measured in millimeters.

The primary outcome was the relation between the AVD measured using transperineal ultrasound and the final diagnosis of the extent of the laceration. Secondary outcomes were the relation between the AVD and the palpated perineal thickness and the relation between the AVD and midwife’s initial diagnosis.

The Regional Ethics Review Board in Linköping approved the study (Dnr 2014/248–31).

### Statistics

Statistical analysis was undertaken with SPSS Statistics, version 24 (IBM, Armonk, NY, USA). ANOVA statistical methods were used in calculations concerning characteristics of the study population, AVD and external sphincter injury and concerning the AVD and initial palpation thickness. For categorical data, considering maternal and obstetric characteristics, the chi-square test was used. Logistic regression analysis was used in calculations concerning AVD as a continuous variable and external sphincter injury. As post hoc test, Bonferroni was used. Sensitivity, specificity and predictive values for AVD were calculated using cross tabulation.

## Results

During the 16-month inclusion period, 150 women with a suspected perineal laceration of degree 2 or 3 were enrolled. None of the women in the study population had an episiotomy, which reflects the standard care at the delivery unit.

Ultimately, after the obstetrician’s examination, the women were diagnosed with lacerations of degree 2 (*n* = 121) or degree 3 (*n* = 29).

External sphincter injury was associated with a shorter measured AVD. In the group ultimately diagnosed as having an external sphincter laceration, the mean AVD was 11.6 mm (95% CI 9.3–13.8). In the group ultimately diagnosed with a second-degree laceration, the mean AVD was 17.8 mm (95% CI 16.9–18.7); thus, the mean AVD difference between the second- and third-degree lacerations was 6.2 mm (95% CI 4.1–8.4, *p* &lt; 0.001). When assessed as a continuous variable, AVD was also inversely correlated with an external sphincter laceration (*p* &lt; 0.001). The shorter the AVD is, the higher the likelihood of an external sphincter injury.

Retrospectively, based on knowledge from the present study of potential positive/negative test results, we calculated the sensitivity, specificity and predictive value.

Using an AVD cutoff of &gt; 20 mm, the sensitivity for sphincter injury was 96% [28/(28 + 1)] and the specificity 25% [30/(91 + 30)]. The positive predictive value was 0.23, and the negative predictive value was 0.97.

The measured AVD was shorter in the midwife-assessed third-degree lacerations than in the second-degree lacerations. The mean AVD in the group with probable second-degree laceration (*n* = 85) was 18.8 mm (95% CI 17.8–19.8, SD 4.5 mm), in suspected third-degree laceration (*n* = 33) it was 15.7 mm (95% CI 13.7–17.7, SD 5.6 mm), and in probable third-degree laceration (*n* = 32) it was 11.8 mm (95% CI 9.7–13.9, SD 5.8 mm) (*p* &lt; 0.001). Comparing the mean anovaginal distance between probable second-degree lacerations and suspected third-degree lacerations, the difference was 3.1 mm (95% CI.6–5.6) (*p* = 0.01). Between probable second-degree lacerations and probable third-degree lacerations, the difference was 7.0 mm (95% CI 4.4–9.5) (*p* &lt; 0.001), and between suspected third-degree lacerations and probable third-degree lacerations, the difference was 3.9 mm (95% CI 0.8–6.9) (*p* = 0.008).

**Fig. 3:**
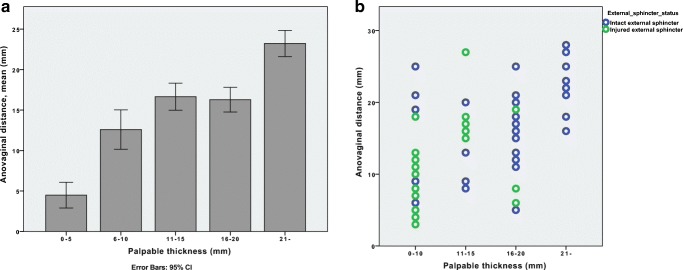
**a** and **b** The palpated perineal thickness and corresponding anovaginal distance (AVD) immediately after delivery (**a**) and considering the ultimately diagnosed external sphincter status (**b**)

The bidigital palpation was classified in six subclasses, palpation thickness 0–5 mm, 6–10 mm, 11–15 mm, 16–20 mm, and 21 mm and above. The AVD differed significantly in the palpation subgroups, as seen in Fig. 3a. One external sphincter injury was found when the AVD was &gt; 20 mm (Fig. 3b). As the palpation thickness was estimated to be &lt; 15 mm, it was considered an outlier.

## Discussion

This prospective observational study evaluated the diagnostics of postpartum perineal laceration by immediate transperineal ultrasound of the AVD in relation to the degree of the tear and bidigital palpation of the perineal thickness.

The main result was that women with an ultimately confirmed diagnosis of external sphincter injury had a shorter AVD compared with women with a perineal laceration without external sphincter injury. The palpatory perineal thickness measured by the midwife correlated well with the transperineal ultrasound measurement of AVD.

To our knowledge, a relation between the AVD and obstetricians’ subsequent classification of the obstetric injury has not been previously evaluated in the acute phase before primary suturing.

This study has certain strengths and limitations. A strength is that it evaluated clinical standard care at a delivery ward. In standard care in Sweden, the midwife is the initial examiner of perineal lacerations. This initial assessment determines the subsequent treatment pathway of the laceration. Three main pathways clinically relevant for excluding or establishing the presence of an anal sphincter injury (“probable degree 2,” “suspected degree 3″ or “probable degree 3”) also determined the groups in the present study. This design may facilitate the applicability of the findings in a clinical setting.

Another strength is that the obstetrician measuring the AVD was not informed about the midwife’s initial assessment of which study group the woman belonged to. The relation between the midwife’s initial assessment and AVD is therefore less likely to be biased. Two persons examined the perineal laceration by two separate methods, bidigital palpation versus transperineal ultrasound, unaware of each other’s results. This study design made it possible to relate the results of one method to the other.

One possible limitation could be the number of missing potential study subjects. Inclusion or not of study subjects was determined by the actual workload at the unit, and a subjective bias such as a complicated delivery might have been taken into account when including women in the study. The gestational week of delivery was slightly but significantly different in the study population compared with the whole target population. This could be explained by the fact that none of the women delivering before the 34th gestational week and fulfilling the inclusion criteria had been included in the study. Studying the circumstances directly after delivery, such as the diagnostics of perineal lacerations, carries challenges as engaged and competent personnel need to be ready 24/7 of and there is the ethical aspect of including women who have just recently undergone a complicated delivery. The special conditions for recruiting pregnant women in research has previously been described by van Delft [16].

It is possible that the obstetricians’ diagnosis of the perineal laceration could have been influenced by being aware of the recently measured AVD. At the time of the study, the examiners had no knowledge about a potential relation between the AVD and presence of external anal sphincter injury. Because of the results of this study, in future studies it would be preferable for independent investigators to perform the different measurements.

To our knowledge, there are no earlier studies examining the relation between the AVD measured by transperineal ultrasound and the occurrence of external anal sphincter injury before suturing the perineal laceration. Örnö et al. [7] studied the identification of several anatomical perineal structures and the interobserver agreement immediately after delivery by transvaginal ultrasound using a biplane endoscope probe. This study showed relatively poor agreement between the observers considering assessing the external sphincter, but the results implied that ultrasound might be helpful in evaluating the extent of perineal lacerations before repair. Measuring the AVD might not be considered as complex as identifying certain specific anatomical structures in the perineal area and has a short learning curve; in a previous study it has shown high interobserver agreement [9].

Endoanal ultrasound improved the diagnosis of anal sphincter tears and reduced the risk of severe fecal incontinence 3 months postpartum [3]. The Cochrane Report on ultrasound related to sphincter injury [17] is also based solely on this study. The report stated that there is some evidence regarding endoanal ultrasound before perineal repair, but suggested further studies should be performed on detection rates, cost analysis and training in the method before routine use of endoanal ultrasound postpartum can be recommended in clinical practice.

A shortcoming of the modality of endoanal ultrasound is, as implied by the Cochrane Report, that it is not practically viable in the acute setting at a delivery ward because of equipment and competence restraints. Comparisons between the modalities of endoanal and the clinically more accessible transperineal ultrasound are therefore important. Stewart et al. [14] used both a side-firing transrectal probe and an end-firing vaginal probe transvaginally and stated that the accuracy of the transvaginally method was equivalent to endoanal ultrasound considering the anal sphincter. A study by Roos et al. [10] comparing endoanal ultrasound to a method technically similar to the measurement of AVD in our study showed low sensitivity (30%) in detecting external sphincter injuries, but high specificity of 95%. Transperineal ultrasound findings on the day of delivery are also related to long-term symptoms [6].

As such, measuring AVD with transperineal ultrasound is a useful method for screening in the acute phase and a tool for identifying normal anatomy. In those women where a normal anatomy cannot be identified, the clinician’s awareness of external sphincter injuries should be increased.

The correlation between a bidigital examination of the external sphincter thickness and transperineal ultrasound of the perineal length was studied by Shobeiri et al. directly after primary repair of an external anal sphincter laceration [13]. The results, which showed good correlation between the physical and ultrasound examinations, corresponded well with the results in this study where the comparison was made directly after childbirth, before suturing. Our results are promising, and further studies with more defined palpation values would be valuable.

## Conclusion

This study showed that AVD measurements were associated with the final diagnosis of the perineal laceration as a short ultrasound AVD postpartum implied a higher risk of external sphincter injury. It also showed a relation between the palpatory assessment of the perineal thickness and the ultrasound-measured AVD.

Based on the results of this study, it is suggested that measuring AVD with transperineal ultrasound with a vaginal probe can contribute information about the degree of laceration before primary suturing. To confirm these new findings, further studies are needed. We suggest that a short AVD could be a warning sign in clinical practice in the delivery room and should increase awareness when further examining the perineal laceration before suturing.

## Electronic supplementary material

Table S1 (DOCX 8 kb)

Fig. S1 (DOCX 56 kb)
