# Supplementary material for: Anovaginal distance and obstetric anal sphincter injury: a prospective observational study
Source: Int Urogynecol J. 2018 Dec 10;30(6):939–44. doi: 10.1007/s00192-018-3838-5 (PMC6511353; doi:10.1007/s00192-018-3838-5)
Supplement: Supplementary file 1 — (DOCX 8 kb) [file 192_2018_3838_MOESM1_ESM.docx]

Table S1. Maternal and Obstetrical characteristics of the study population between initial assessment groups.

* Pearson Chi-square test, df 2

|  | Probable grade 2  (n=82) | Suspected grade 3  (n=31) | Probable grade 3  (n=33) | p-value |
| --- | --- | --- | --- | --- |
| Age | 29.9 (3.82) | 28.7 (4.35) | 30.6 (4.90) | 0.18 |
| BMI mean (SD) | 24.4 (4.41) | 24.0 (3.79) | 23.8 (3.78) | 0.75 |
| Gestational week | 39.9 (1.32) | 39.9 (1.19) | 40.1 (1.82) | 0.89 |
| Smoking | 0 | 2 | 0 | na |
| Living with a partner | 79 | 29 | 32 | 0.73* |
