# Supplementary material for: Anovaginal distance and obstetric anal sphincter injury: a prospective observational study
Source: Int Urogynecol J. 2018 Dec 10;30(6):939–44. doi: 10.1007/s00192-018-3838-5 (PMC6511353; doi:10.1007/s00192-018-3838-5)
Supplement: Supplementary file 2 — (DOCX 56 kb) [file 192_2018_3838_MOESM2_ESM.docx]

Table S1. Flow-chart of the inclusion process.

2574 women having their first child

*Target population*

Was not asked N=1107

Rejected inclusion

N=142

excluded

excluded

Examination with perineal ultrasound

N=150

Suspected grade 3-laceration

N=33

Probable grade 3-laceration.

N=32

N=243

Probable grade 2-laceration

N=85

Study population

N=150

Obstetric laceration grade 2-3

N=1605

Obstetric laceration grade 1 or 4.

N=969

Met exclusion criteria N=206

N=1043

Not included
